# Posttraumatic Stress Disorder and Not Depression Is Associated with Shorter Leukocyte Telomere Length: Findings from 3,000 Participants in the Population-Based KORA F4 Study

**DOI:** 10.1371/journal.pone.0064762

**Published:** 2013-07-03

**Authors:** Karl-Heinz Ladwig, Anne Catharina Brockhaus, Jens Baumert, Karoline Lukaschek, Rebecca T. Emeny, Johannes Kruse, Veryan Codd, Sibylle Häfner, Eva Albrecht, Thomas Illig, Nilesh J. Samani, H. Erich Wichmann, Christian Gieger, Annette Peters

**Affiliations:** 1 Institute of Epidemiology II, Helmholtz Zentrum München—German Research Center for Environmental Health, Neuherberg, Germany; 2 Department for Psychosomatic Medicine and Psychotherapy, Klinikum Rechts der Isar, Technische, Universität München, Munich, Germany; 3 Institute of Genetic Epidemiology, Helmholtz Zentrum München—German Research Center for Environmental Health, Neuherberg, Germany; 4 Department of Medicine I, University Hospital Grosshadern, Ludwig-Maximilians University Munich, Munich, Germany; 5 Department for Psychosomatic Medicine and Psychotherapy in the University Hospital of the University of Giessen, Giessen, Germany; 6 Department of Psychiatry, University Medical Center, Georg August University Goettingen, Goettingen, Germany; 7 Research Unit of Molecular Epidemiology, Helmholtz Zentrum München—German Research Center for Environmental Health, Neuherberg, Germany; 8 Hannover Unified Biobank, Hannover Medical School, Hannover, Germany; 9 Department of Cardiovascular Sciences, University of Leicester, Glenfield Hospital, Leicester, United Kingdom; 10 Leicester NIHR Biomedical Research Unit in Cardiovascular Disease, Glenfield Hospital, Leicester, United Kingdom; 11 Institute of Epidemiology I, Helmholtz Zentrum München—German Research Center for Environmental Health, Neuherberg, Germany; University of Nebraska Medical Center, United States of America

## Abstract

**Background:**

A link between severe mental stress and shorter telomere length (TL) has been suggested. We analysed the impact of Posttraumatic Stress Disorder (PTSD) on TL in the general population and postulated a dose-dependent TL association in subjects suffering from partial PTSD compared to full PTSD.

**Methods:**

Data are derived from the population-based KORA F4 study (2006–2008), located in southern Germany including 3,000 individuals (1,449 men and 1,551 women) with valid and complete TL data. Leukocyte TL was measured using a quantitative PCR-based technique. PTSD was assessed in a structured interview and by applying the Posttraumatic Diagnostic Scale (PDS) and the Impact of Event Scale (IES). A total of 262 (8.7%) subjects qualified for having partial PTSD and 51 (1.7%) for full PTSD. To assess the association of PTSD with the average TL, linear regression analyses with adjustments for potential confounding factors were performed.

**Results:**

The multiple model revealed a significant association between partial PTSD and TL (beta = −0.051, p = 0.009) as well as between full PTSD and shorter TL (beta = −0.103, p = 0.014) indicating shorter TL on average for partial and full PTSD. An additional adjustment for depression and depressed mood/exhaustion gave comparable beta estimations.

**Conclusions:**

Participants with partial and full PTSD had significantly shorter leukocyte TL than participants without PTSD. The dose-dependent variation in TL of subjects with partial and full PTSD exceeded the chronological age effect, and was equivalent to an estimated 5 years in partial and 10 years in full PTSD of premature aging.

## Introduction

Telomeres are tandem repeats of hexamers (TTAGGG) at the ends of chromosomes that protect against spontaneous DNA damage and thus preserve genomic integrity. Due to the so-called “*end-replication problem*” [1] which leaves a small region at the end of the chromosome uncopied during mitosis, and further mechanisms inherent to the genomic replication machinery [2], telomeres progressively shorten with each cell replication in human somatic cells. However, telomere attrition is also promoted by multiple conditions which lead to oxidation and inflammation [3].

Previous studies showed substantial associations of TL with sex and age [4] as well as with obesity [5], [6], current smoking [7], alcohol consumption [8], physical inactivity [9], hypertension [10], cholesterol [11] and a variety of chronic disease conditions such as metabolic diseases or cancer [12]–[14]. Additionally, sustained states of severe mental stress and adverse affective conditions have also been associated with shorter TL. Among the first investigations were two studies which observed shorter TL in premenopausal women with a long duration of high perceived stress due to caring for chronically ill children [15] or Alzheimer's disease patients [16]. However, no significant association of perceived stress and TL was found in a large population-based sample of sisters of breast cancer patients [17]. Stress exposure during intrauterine development [18] and in early postnatal life (infancy and childhood) has been positively associated with shorter TL [19]–[21], although a negative finding was also reported [22]. The effect of childhood adversities on TL was stronger than suffering from anxiety disorder in adult life [23]. Recently, in a 5-year follow-up study, Shalev et al. [24] reported shorter telomeres in children who experienced violence.

Depression has been associated with shorter telomeres in clinical populations with severe and long acting disease conditions [10], [25]–[27]. In particular, depression was also associated with shorter telomeres in patients with coronary heart disease even after adjustment for somatic risk factors [28]. Severe levels of work-related exhaustion in a Finnish sample of 2,911 study participants proved to be significantly associated with shorter leukocyte telomere length, compared to non-affected counterparts [29]. Conflicting evidence comes from studies which could not confirm an association of TL with depression severity [30], possibly due the use of screening instruments instead of clinical interviews when assessing depression [17], [27], [31]–[33]. In a study including 18 non-medicated patients with major depression [26], depression per se was not associated with TL but lifetime depression exposure correlated significantly with shorter telomeres. Recently, a prospective study showed anxiety disorder to predict later telomere shortening while depression was not predictive [34]. High phobic anxiety was related to lower leukocyte TL in a large scale population-based study in women [35], while general anxiety was associated with TL only in the older half of a large scale study group of Finish anxiety disorder patients [23]. Anxiety was not associated with TL in participants of the EPIC-Norfolk study group [32].

Taken together, the evidence suggests that TL attrition apparently requires long time periods or substantial impact in terms of a dose/response relationship to shorten TL significantly. Among mental health disease conditions, posttraumatic stress disorder (PTSD) is one of the most disabling and severe conditions, with accumulating evidence that chronic PTSD may moderate the link of adverse sustained mental health conditions to secondary negative health outcomes such as cardiovascular and metabolic diseases [36]–[39].

Thus, PTSD may be a candidate to influence TL attrition although a first investigation in 43 adults with chronic PTSD (n = 18 with multiple categories of childhood trauma) and 47 control subjects (none with multiple categories of childhood traumata) revealed shorter TL only in those trauma patients with childhood traumata [20]. In an exploratory study with female rape victims from South Africa, nine of participants (15%) were diagnosed with PTSD three months after the trauma. For these victims, a marginally significant association was evident between TL (measured at baseline) and PTSD which led the investigators to suggest that rape victims with pre-existing shorter relative LT might be more susceptible and thus less resilient to developing a major depressive disorder (MDD) or PTSD related to rape trauma.

The aim of the present investigation was to analyse the association of PTSD with alterations of TL in a sample drawn from the general population. We also hypothesised that there is a dose-dependent association of shorter TL with severity of PTSD. Assuming that the association of PTSD with TL might be affected by other variables, we selected a priori chosen set of potential confounders.

## Materials and Methods

### Study design and population

Data were derived from the population-based KORA (Cooperative Health Research in the Region of Augsburg) F4 study (2006–2008), a follow-up study of the KORA S4 survey conducted in 1999–2001. The study area is located in southern Germany, and comprises the city of Augsburg and two surrounding counties with approximately 600,000 inhabitants, living in a mixed urban and rural area whose demographic and socioeconomic characteristics roughly reflect those of the average central European population in general. Written informed consent was obtained from each participant. The study was approved by the local ethics committee and performed in the KORA Study Center, Augsburg. All participants filled in a self-administrated questionnaire and underwent a standardized personal interview and an extensive medical examination. All procedures were subjected to a constant quality assessment. All data used in the present analyses were drawn from the KORA F4 study.

The study design, sampling method and data collection have been described in detail elsewhere [40]. Briefly, from 6,640 eligible subjects aged 25–74 years in the baseline *Survey* 4 (S4) randomly selected from the population, a total of 4,261 participated at baseline examination. For the 7-year-follow-up *F4 Study* (2006–2008) with subjects now in the age range of 32–81 years, loss to follow-up was due to subjects who had died (n = 176; 4%), lived too far outside the study region or were lost to follow-up (n = 206; 5%), or had demanded deleting their address data (n = 12; 0.2%). Of the remaining 3,867 eligible subjects, 174 could not be contacted, 218 were unable to participate because they were too ill, and 395 were unwilling to participate, giving a response rate of 79.6% (n = 3,080).

For the present study, a number of 80 subjects with missing information on PTSD, telomere length or any of the covariates under concern were excluded from the analyses. Therefore, the study population consisted of 3,000 individuals (1,449 men and 1,551 women). An overview about descriptive data is given in Table 1.

**Table 1:** Distribution of characteristics of the study population (n = 3,000) by PTSD status.

	PTSD status			
	no PTSD (n = 2,687)	partial PTSD (n = 262)	full PTSD (n = 51)	p value
*Age (years)*	*56.5±13.4*	*52.5±10.6*	*54.5±11.8*	*<0.001*
*Male sex (%)*	*49.5*	*38.2*	*37.3*	*<0.001*
*Low educational level (%)*	*59.0*	*57.6*	*64.7*	*0.642*
*Living alone (%)*	*24.1*	*29.4*	*35.3*	*0.036*
*BMI (kg/m^2^)*	*27.6±4.8*	*27.4±5.1*	*28.1±5.7*	*0.897*
*Current smoking (%)*	*17.8*	*21.4*	*9.8*	*0.108*
*Alcohol consumption (%)*				*0.360*
*No*	*30.1*	*30.2*	*35.3*	
*Moderate*	*52.7*	*49.6*	*41.2*	
*High*	*17.2*	*20.2*	*23.5*	
*Physical inactivity (%)*	*45.7*	*42.7*	*47.1*	*0.646*
*Actual hypertension (%)*	*31.7*	*24.4*	*27.5*	*0.045*
*TC/HDL-C*	*4.09±1.18*	*4.04±1.20*	*4.02±1.21*	*0.484*
*History of chronic diseases*	*16.8*	*22.9*	*19.6*	*0.041*
*Depression (PHQ-9) (%)* *	*3.9*	*13.0*	*5.9*	*<0.001*
*Depressed mood/exhaustion (DEEX) (%)* *	*18.4*	*55.0*	*56.9*	*<0.001*

*Distributions are presented as means ± standard deviation for continuous, p values from χ^2^ test or F test for association with PTSD,*

* *only n = 2,528.*

### Measurement of posttraumatic stress disorder (PTSD)

Posttraumatic stress disorder is characterized by a constellation of distressing and/or impairing symptoms that can arise as a response to a stressful event or situation of an exceptionally threatening or catastrophic nature, which is likely to cause pervasive distress in almost anyone. According to ICD-10 [41], the first criterion for a diagnosis of PTSD to be made requires that an individual be exposed to a traumatic event (Criterion A). To assess criterion A in KORA F 4, the Posttraumatic Diagnostic Scale (PDS) [42] containing a list of 11 extremely stressful events was given as well as an open question about other traumatic events. Core PTSD symptoms encompass the re-experiencing of the trauma in intrusive memories, flashbacks or nightmares (Criterion B), avoidance of activities and situations reminiscent of the trauma (Criterion C), and a state of hyperarousal with hypervigilance, an enhanced startle reaction and insomnia (Criterion D). The impact of event scale (IES) by Horowitz *et al.* [43] was applied to assess the intrusion and avoidance criteria (B, C). To qualify for the hyperarousal criterion (D), any two of the following symptoms had to be met: Irritability, inner tension, uneasiness, difficulty to concentrate, hyperhidrosis and insomnia (assessed by a face to face interview).

Subjects who met criteria A-D were counted as ‘full PTSD’ cases. Subjects who met criterion A and any one or two of the criteria B-D were counted as ‘partial PTSD’ cases [44]. All other participants were defined as ‘no PTSD’ subjects.

### Measurement of telomere length (TL)

The telomere length in the present KORA F4 study data was measured using a quantitative PCR-based technique [45], which expresses the average telomere length as the ratio (T/S) of the telomere repeat copy number (T) to a single copy gene (S). In order to standardise the measurements across PCR plates the T/S ratio is measured relative to a standard DNA used within each assay (genomic DNA from the K562 cell line). The method is described in detail elsewhere [46]. The distribution of the average telomere length in the present study followed approximately a normal distribution.

### Measurement of covariates

*Educational level* was assessed by dichotomizing the variable in less than 12 years of education, defined as low education, and 12 or more years of education, defined as high education. *Living alone* was assessed by asking subjects whether they lived alone, lived with a partner, were married, living together, married, but living separately, divorced or widowed. Being married and living with the partner as well as living together with a partner without being married were defined as ‘living not alone’. Living alone, being married, but living separately, being divorced or widowed were defined as ‘living alone’.

*Body mass index* (BMI) was calculated as weight in kilograms divided by the height in meters squared. Obesity was defined as having a BMI ≥ 30 kg/m^2^. *Current smoking* was defined as currently smoking at least one cigarette per day or occasionally smoking. *Alcohol consumption* was measured continuously (g/day) and classified into the three categories no, moderate (>0–39.9 g/day for men, >0–19.9 g/day for women) and high (≥40 g/day for men, ≥ 20 g/day for women). To assess *physical inactivity*, participants were classified as ‘active’ during leisure time if they regularly participated in sports for at least 1 hour per week and ‘inactive’ else. *Actual hypertension* was defined as blood pressure of ≥160/95 mm Hg or current use of hypertensive medication. *Total cholesterol* (TC) and *high density lipoprotein cholesterol* (HDL-C) were measured in mg/dl by enzymatic methods (CHOD-PAP, Boehringer Mannheim, Germany) and combined by dividing TC by HDL-C denoted as *TC/HDL-C*. Assessment of a *history of chronic diseases* was based on self-report and was defined as having a history of myocardial infarction, diabetes, stroke or cancer.

*Depression* was assessed by a short version of the Patient Health Questionnaire (PHQ-9) that captures depressive symptoms and was administered in the personal interview [47]. *Depressed mood/exhaustion* was assessed by the DEpression and EXhaustion subscale (DEEX scale) from the von Zerssen symptom checklist assessed in a self-administered questionnaire [48], [49].

### Statistical Analysis

Univariate associations between categorical variables were assessed by the *χ2* test; for group differences in continuous variables, the *F* test was applied. Pearson correlation was used to estimate linear correlations between continuous variables.

To assess the association of PTSD with the average telomere length, linear regression analyses with different adjustments for potential confounding factors were performed using PTSD (no, partial, full PTSD) as exposure variable with no PTSD as reference category and TL as outcome variable. First, to assess the relationship of partial PTSD or full PTSD with the average telomere length compared to subjects with no PTSD, a basic linear regression model (model 1) with adjustment for age, sex and BMI was applied. Second, to assess if this relation was confounded by further covariates, a multiple linear regression model (model 2) with additional adjustment for smoking status, alcohol consumption, physical inactivity, actual hypertension, TC/HDL-C and history of chronic diseases was estimated. These additional covariates might have an impact on telomere length and might thus be taken into account when assessing the association of PTSD and TL independent of potential confounding factors.

Furthermore, to analyse whether controlling for depressive disorders might affect the PTSD-TL association, the covariates depression (PHQ-9) and depressed mood/exhaustion (DEEX) were included additionally to the covariates of model 2 in a linear regression. This analysis could only be applied to 2,528 subjects due to missing values for the two depressive disorder covariates, since subjects aged ≥72 years were excluded from the psychosomatic examinations. The R^2^ was used to assess the goodness of model fit.

P values below 0.05 were considered to be significant. All evaluations were performed with the statistical software SAS for Windows, Version 9.2.

## Results

### Description of the study population by PTSD symptoms

In the total study sample, 2,687 (89.6%) subjects were free of PTSD while 262 (8.7%) subjects suffered from partial PTSD and 51 (1.7%) from full PTSD. A descriptive overview of the study population, stratified for the three groups (healthy, partial PTSD and full PTSD) is given in Table 1 . Compared to subjects with no PTSD, partial and full PTSD participants were likely to be younger, to be female, and to be more often living alone than their respective counterpart. Concerning classical risk factors (BMI, smoking status, alcohol consumption, physical inactivity, TC/HDL-C, chronic diseases), no significant univariate differences between PTSD groups were observed except for actual hypertension, which was more frequent among non-PTSD participants and for history of chronic diseases, which was more frequent among partial and full PTSD participants. Moreover, univariate associations with PTSD were found for depression and for depressed mood/exhaustion.

### Distribution of the average telomere length

Significantly longer telomeres were measured in women compared to men (1.90±0.32 versus 1.79±0.33, p value<0.001). With increasing age, the telomere length decreased in individuals (Pearson correlation coefficient −0.40, p value<0.001). Compared to no PTSD (1.85±0.33), the mean TL was almost equal in partial PTSD (1.85±0.29) and lower in full PTSD (1.78±0.29, p value) reaching no significance in unadjusted analyses (p values 0.99 and 0.14). However, due to the strong correlation between PTSD and age, the association of PTSD and TL association was significant when the strong TL-age correlation was taken into account by adjusting for age (p values 0.043 and 0.027).

### Association of PTSD with the average telomere length

**Figure 1:**
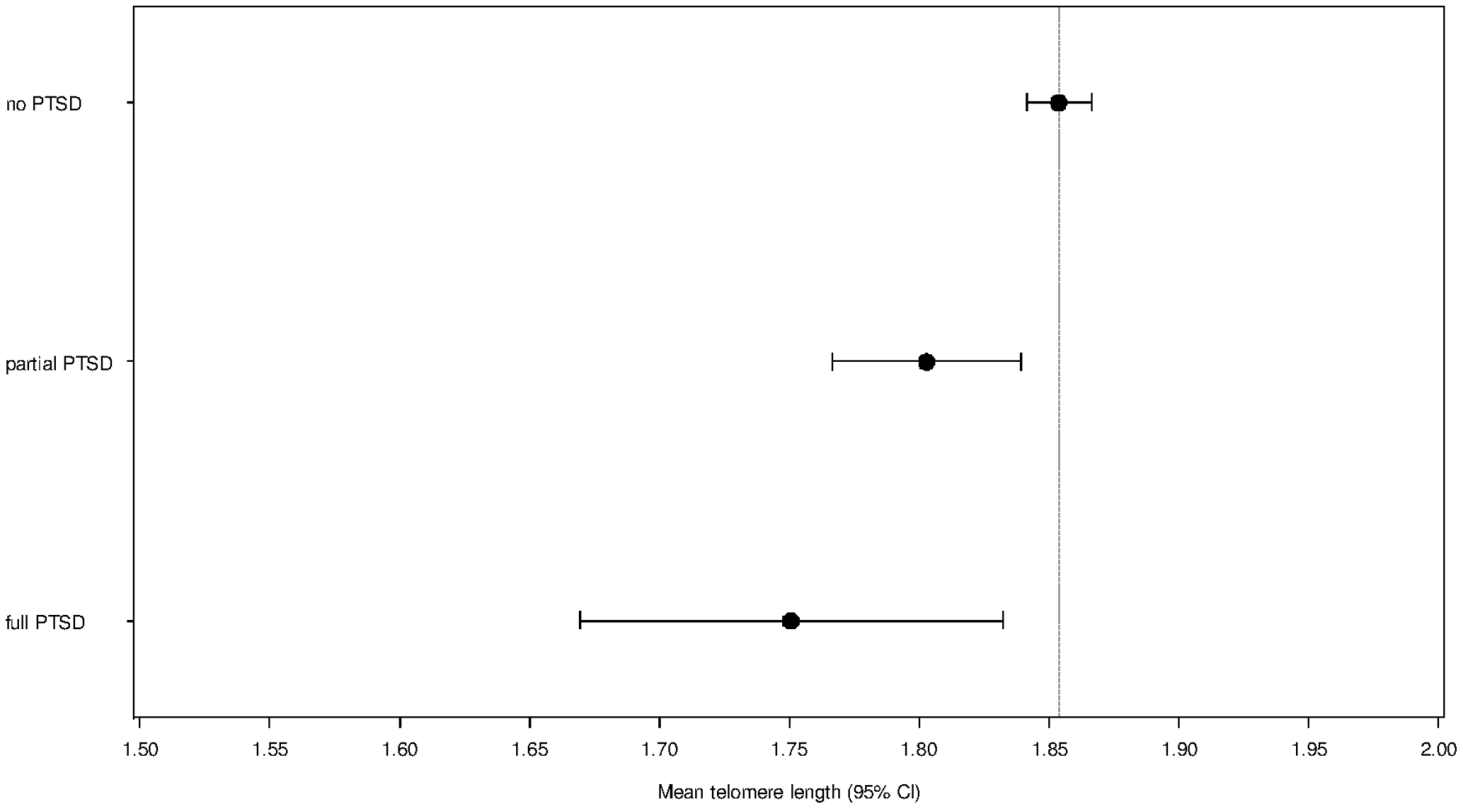
Mean telomere length of study participants, stratified by PTSD status and adjusted for age, sex, BMI, smoking status, alcohol consumption, physical inactivity, actual hypertension, TC/HDL-C and history of chronic diseases. *Vertical reference line denotes mean TL for no PTSD*.

The association between PTSD and average TL was evaluated by linear regression models shown in Table 2. The basic linear regression model 1 (adjusted for age, sex and BMI), revealed a significant association between partial PTSD and TL (beta = −0.050, p = 0.010) as well as between full PTSD and TL (beta = −0.104, p = 0.013). These findings indicate that participants with partial or full PTSD had significantly shorter TL than participants with no PTSD. These associations were confirmed in a multiple-adjusted model shown by very similar estimates for partial PTSD (beta = −0.051, p = 0.009) and full PTSD (beta = −0.103, p = 0.014) (model 2). In this model, besides age and sex ( p values<0.001), only BMI had a significant association with telomere length in the present data set (p value 0.002) This additional adjustment revealed almost equal differences in the mean telomere length for subjects with partial or full PTSD compared to no PTSD subjects as model 1. Figure 1 shows the mean telomere length as drawn from model 2. R^2^ values were 0.187 for model 1 and 0.188 for model 2.

**Table 2:** Association of PTSD with telomere length estimated by linear regression.

	*Model 1*		*Model 2*	
	*Beta coefficient (95% CI)*	*p value*	*Beta coefficient (95% CI)*	*p value*
*no PTSD*	*ref.*	*-*	*ref.*	*-*
*partial PTSD*	*−0.050 (−0.088–−0.012)*	*0.010*	*−0.051 (−0.089–−0.013)*	*0.009*
*full PTSD*	*−0.104 (−0.186–−0.022)*	*0.013*	*−0.103 (−0.185–−0.021)*	*0.014*

*1) Model 1 was adjusted for age, sex and BMI.*

*2) Model 2 was adjusted for age, sex and additionally for smoking status, alcohol consumption, physical inactivity, actual hypertension, TC/HDL and history of chronic diseases.*

*R^2^: 0.187 (model 1), 0.188 (model 2).*

Controlling additionally for depression and depressed mood/exhaustion in 2,528 participants gave comparable effect sizes with beta estimate −0.056 for partial PTSD and −0.107 for full PTSD which remained significant (p value 0.007 and 0.014, respectively). It is of note that, both, depression and depressed mood/exhaustion, were not significantly associated with TL in this multiple model (p values 0.51 and 0.44). Even in a model adjusted for age and sex only, PTSD had a significant association with TL (p value 0.002) but none of the depressive symptoms variables.

Since the estimates for partial or full PTSD (beta for partial PTSD = −0.051, beta for full PTSD = −0.103) was found to be about five and ten times as strong as the age estimates per year (beta for age = −0.010), it can be estimated that the biological age of participants with partial PTSD exceeded their chronological age by approximately 5 years and in participants with full PTSD by 10 years.

## Discussion

PTSD is a mental disease condition with a sustained psychological response pattern to a major stressful traumatic event. Subjects suffering from PTSD are unable to recover from the impact of such a stressor by displaying symptoms encompassing the key clusters of intrusion (unintended re-experiencing the stressor event), avoidance (of any reminder of the stressor) and hyperarousal (e.g. startle and other forms of unspecific hyper- sympathetic responsiveness). To the best of our knowledge, this is the first study to show an association of PTSD and shorter telomere length (TL) in a population-based sample. The finding remained stable after adjustment for classical risk factors. Interestingly, depression and depressed mood/exhaustion as major psychopathological conditions contributing to the clinical picture of PTSD [50] additionally did not weaken the association between PTSD and TL.

Giving the strong genetic determination of telomere length [46], the risk factor adjusted association between PTSD and TL is a remarkable finding – although in line with earlier investigations which have shown associations of TL with long acting psychological stress conditions and sustained negative affectivity (major depression, anxiety) [10], [19]–[21], [25]–[29], [34].

The index population of the present investigation was drawn from a random population-based sample. Up to now, the impact of adverse mental health conditions on TL was particularly shown in clinical studies with severe conditions as evidenced in duration, chronicity and symptom burden. Surprisingly, population-based studies with high numbers of study participants involved, yielded insignificant findings so far. For example, Surtees et al. [32] failed to find an association of TL with (lifetime and past-year) major depression, general anxiety disorder and depressive symptoms in an investigation with more than 4,000 women included, aged 41–80 years from the EPIC-Norfolk Population Study.

The present investigation suggests a dose-response effect of TL with a smaller TL alteration for partial PTSD compared to full PTSD. It indicates that the effect of stress is most clearly seen when occurring in a cumulative way, which is typically the case in PTSD sufferers who often through flash-backs or intensive memories re-experience their traumatic life event perpetually over the course of their disease process. The distinct and graded response pattern of TL shortening in partial and full PTSD cases also confirms the clinical relevance of distinguishing the subdivision of PTSD [51], [52].

Patients with PTSD generally cluster within further mental health anomalies [53]. Notably, adjustment of depression and depressed mood/exhaustion (both highly associated with PTSD in the present analysis) did not influence the strength of the association between PTSD and TL which may point to an independent pathway between these conditions. Furthermore, depression and depressed mood/exhaustion were not associated with telomere length in the present study which is in consent with previous studies [10], [25]–[27] but disagrees with other findings [17], [31], [32].

The present study was not designed to reveal possible mechanisms linking PTSD and TL. However, it is generally acknowledged that oxidation and inflammation are likely to be major contributors to telomere shortening. Consequently, conditions which are known to be highly related to these conditions impact TL substantially. Emerging awareness considers inflammation as a psychophysiological biomarker for chronic psychosocial stress and recent investigations have provided evidence for low-grade systemic pro-inflammatory activity in patients with PTSD [54]. Interestingly, Wikgren et al. recently showed that a hypocortisolemic state was associated with shorter TL [27]. Hypocortisolism has been described most consistently in PTSD [55].

In contrast to the chronological age, telomere length has been suggested to be a useful marker of biological age. This is, however, largely a theoretical assumption with only little proven evidence, and even more questionable in light of recent findings from a limited number of prospective studies which have shown that telomere length may shorten, remain stable or even increase in length. Thus, it remains undecided [56] whether shorter telomeres in PTSD patients in the present study reflect a pathological condition or are benign or inconsequential markers [57]. Hoen et al. recently followed 952 patients with coronary heart disease over a 5-year observation period and found that depression at baseline was associated with a 32% greater odds of lengthening [28]. Willeit et al., who demonstrated a significant inverse correlation between short baseline TL and cancer incidence and mortality, concluded that it is tempting to speculate that “…cells with a critically short TL may under certain circumstances reactivate the enzyme telomerase to escape from cell senescence and thereby facilitate malignant transformation” [58].

The major advantage of the present investigation is the unbiased access to the index population through a large scale population-based study. However, the present study has also several limitations. The design of the study is cross-sectional and thus sensitive for residual confounding. However, longitudinal analysis of subjects without PTSD at baseline remains a challenge (among others due to low incidence rates after experiencing a traumatic event [44]). No data is available on the exposure time of suffering from the aftermath of a traumatic event. For some selected traumata (dealing with childhood adversity) a lifelong exposure is likely.

For telomere measurement, only one technique was applied which might affect the present findings. The cell type used here were blood leukocytes. Although most research in telomere biology has been undertaken with white blood cells, one has to acknowledge that leukocyte populations are a heterogeneous mixture of cells including neutrophils, T cells, monocytes, B cells etc., each of which may respond differently to stress [59]. Currently, it is not fully clear whether measurements in leukocytes are representative of the processes that occur in other somatic cells. However, evidence speaks for leukocyte TL as a surrogate for relative TL in other tissues [60].

## Conclusions

The present investigation shows that PTSD is a severe mental stress condition which impairs the basic molecular mechanisms in cell replication. Participants with PTSD had significantly shorter TL than participants without PTSD. The dose-dependent attrition rate of TL in partial and full PTSD cases accounts for the estimation of premature aging of 5 years in partial and 10 years in full PTSD. The specific malignant properties of PTSD causing TL shortening remain to be elucidated. One line of future research should identify those PTSD cases who present high inflammatory burden to evaluate whether TL is more likely among these patients compared to PTSD cases without inflammatory comorbidity. Furthermore, prospective analyses are urgently needed to confirm the present cross-sectional finding.
